# Determining the Relationship Between Change Fatigue and Innovation Climate Among Nurses: A Cross‐Sectional Study

**DOI:** 10.1002/nop2.70820

**Published:** 2026-09-16

**Authors:** Yeter Uslu, Yasemin Hancioğlu Başköy, Gözde Yeşilaydin, Erman Gedikli, Emre Yilmaz

**Affiliations:** ^1^ Department of Health Management, Faculty of Health Sciences Istanbul Medipol University Istanbul Turkey; ^2^ Department of Accounting and Tax, Ünye Vocational School Ordu University Ordu Turkey; ^3^ Department of Health Management, Faculty of Health Sciences Eskişehir Osmangazi University Eskişehir Turkey

**Keywords:** change fatigue, cross‐sectional studies, innovation climate, nurses, organizational change

## Abstract

**Aim:**

To determine nurses' levels of change fatigue and perceived innovation climate and to examine the association between these constructs.

**Design:**

A descriptive, cross‐sectional study.

**Methods:**

A census approach was used among 194 eligible nurses working in two healthcare centres; 186 completed the survey (response rate, 95.9%). Data were collected using a sociodemographic form, the Change Fatigue Scale, and the Innovation Climate Scale. Descriptive comparisons, Pearson correlation, and hierarchical multiple linear regression were conducted.

**Results:**

Mean change fatigue and innovation climate scores were 2.85 (SD 0.77) and 3.21 (SD 0.58), respectively. Higher perceived innovation climate was associated with lower change fatigue (*r* = −0.438, 95% CI [−0.547, −0.314], *p* < 0.001; *r* ^2^ = 0.192). This association remained after adjustment for age, gender, income, work schedule and institutional tenure (*B* = −0.528, 95% CI [−0.711, −0.345], standardized *β* = −0.400, *p* < 0.001).

**Conclusion:**

Perceived innovation climate was inversely associated with change fatigue among nurses. The cross‐sectional design does not establish direction or causality; prospective and intervention studies are needed before concluding that changes to innovation climate reduce fatigue.

**Implications for the Profession and/or Patient Care:**

Nursing managers can use these findings to identify potentially relevant organizational conditions—communication, participation in change, workload and access to innovation resources—that warrant assessment and prospective evaluation.

**Patient or Public Contribution:**

No patient or public contribution.

## 1 Introduction

Healthcare organizations must continually adapt to technological developments, evolving patient expectations and regulatory change. Although organizational change is necessary for service continuity and quality, repeated change initiatives can place additional cognitive and emotional demands on staff. Nurses may be particularly exposed because they provide continuous patient care while adapting workflows, technologies, documentation systems and clinical protocols.

Change fatigue refers to employees' tiredness, strain and diminished capacity to respond when they perceive organizational change as excessive or continuous (Bernerth et al. 2011). It is related to, but conceptually distinct from, burnout: change fatigue is specifically anchored to repeated change demands, whereas burnout is a broader occupational syndrome. Among nurses, change fatigue may coincide with lower motivation, job dissatisfaction and difficulty sustaining engagement during implementation efforts (Abdelwahid and Ata 2022; Elhay et al. 2022).

Innovation climate describes employees' perceptions that their organization supports new ideas, tolerates constructive differences, and provides time, personnel and other resources for innovation. The Turkish nursing version of the Innovation Climate Scale represents support for innovation, low barriers to innovation, and resource provision (Sönmez et al. 2017). A more supportive climate has been associated with innovative work behaviour, psychological empowerment, and job‐related attitudes in nursing and other organizational settings (Baig et al. 2022; Demircioğlu 2023; Lv et al. 2021).

The Job Demands–Resources (JD–R) model provides a conceptual explanation for a possible association between the two constructs (Demerouti et al. 2001). Repeated organizational changes may function as job demands because they require sustained adaptation, whereas autonomy, supervisory support, time and material resources can function as job resources. An innovation‐supportive climate may therefore mark the availability of resources relevant to change. This framework predicts an association between higher perceived resources and lower exhaustion‐related responses, but it does not by itself establish a causal or buffering effect in cross‐sectional data.

Previous nursing research has examined innovation climate mainly in relation to innovative work behaviour, empowerment and leadership (Korku and Kaya 2023; Labrague and Toquero 2023; Lv et al. 2021; Muksoud et al. 2022; Wang et al. 2024). Change fatigue has more often been studied in relation to resilience, job satisfaction and organizational commitment (Abdelwahid and Ata 2022; Elhay et al. 2022). Few studies have quantified the direct association between change fatigue and innovation climate in the same nursing sample, and unadjusted comparisons cannot determine whether the association persists after relevant demographic and work characteristics are considered. This is the specific gap addressed by the present study.

Accordingly, this study aimed to describe nurses' change fatigue and perceived innovation climate, compare scores across selected demographic and work characteristics, and examine their unadjusted and adjusted association. We hypothesized that higher perceived innovation climate would be associated with lower change fatigue.

## 2 Methods

### 2.1 Purpose and Type of Research

This descriptive, cross‐sectional study examined nurses' change fatigue and perceived innovation climate, group differences in these scores, and the association between the two constructs.

### 2.2 Population and Sample of the Study

The target population comprised 194 nurses working in two health practice and research centres affiliated with Istanbul Medipol University. Because the entire eligible population was approached, no sampling procedure was applied. Data were collected from April to June 2022. Of the 194 eligible nurses, 186 returned complete questionnaires (response rate, 95.9%); the other eight declined, were on annual leave, or returned incomplete questionnaires. Analyses therefore included 186 complete cases. Because the study used a census approach, no a priori sample‐size target determined recruitment. A sensitivity power analysis is reported in Section 2.5.

### 2.3 Measures

#### 2.3.1 Change Fatigue Scale

The Change Fatigue Scale is a six‐item, unidimensional measure developed by Bernerth et al. (2011); a Turkish version has demonstrated validity and reliability among healthcare workers (Ekingen and Yıldız 2021). In the questionnaire administered for this study, each item was rated from 1 (strongly disagree) to 5 (strongly agree), and the item mean was calculated; no items were reverse scored. Higher scores indicate greater change fatigue. The five‐point response format used here differs from the seven‐point format in the healthcare‐worker validation study and is addressed as a limitation. Cronbach's alpha was 0.836 in the present sample.

#### 2.3.2 Innovation Climate Scale

The 14‐item Turkish nursing version of the Innovation Climate Scale contains support for innovation (five items), innovation barriers (six items) and resource provision (three items; Sönmez et al. 2017). Items are rated from 1 (strongly disagree) to 5 (strongly agree). The six barrier items were reverse scored as 6 minus the item response before subscale and total means were calculated. Higher total and subscale scores therefore indicate a more supportive climate, fewer perceived barriers, and greater resource provision. Cronbach's alpha was 0.812 for the total scale and 0.886, 0.846 and 0.756 for the three subscales, respectively.

### 2.4 Data Collection

After written informed consent, participants completed the structured questionnaire face to face between April and June 2022. No scale‐item data were missing among the 186 questionnaires included in the analysis.

### 2.5 Statistical Analysis

Means, standard deviations, medians, ranges, counts and percentages were used as appropriate. Skewness and kurtosis values were within ±1.5. Independent‐samples *t*‐tests and one‐way ANOVA were used for group comparisons; Levene's test assessed homogeneity, and Welch ANOVA was used when variances were unequal. Pearson correlation quantified bivariate associations. All tests were two‐sided with *α* = 0.05, and *p* values are reported as < 0.001 rather than 0.000.

Hierarchical multiple linear regression was used with change fatigue as the outcome. Age, gender, income, work schedule and institutional tenure were entered as prespecified demographic and work‐related covariates, followed by innovation climate. Professional tenure was not entered with age because they were highly correlated (*r* = 0.897). Unstandardized coefficients, 95% confidence intervals, standardized *β* for innovation climate, adjusted *R* ^2^, change in *R* ^2^, and incremental Cohen's *f* ^2^ are reported. Residual, influence, homoscedasticity and multicollinearity diagnostics were examined. As a sensitivity check, HC3 heteroscedasticity‐robust standard errors were estimated. All analyses were conducted using Python 3.13.5 with statsmodels 0.14.6 and SciPy 1.17.1.

Because the full eligible population was approached, a sensitivity rather than an a priori recruitment analysis was conducted. Using the fixed‐model multiple‐regression *R* ^2^‐increase framework described for G*Power 3.1 (Faul et al. 2009), *N* = 186, *α* = 0.05, one tested predictor, and seven total predictors provided 80% power to detect an incremental effect of *f* ^2^ = 0.043 or larger.

### 2.6 Ethical Considerations

Ethical approval was obtained before recruitment from the Istanbul Medipol University Non‐Interventional Clinical Research Ethics Committee (approval no. E‐10840098‐772.02‐1544; 4 March 2022). Participants received information about the study and provided written informed consent before participation. Participation was voluntary, and the analysis dataset contained no direct identifiers.

The manuscript was prepared in accordance with the Strengthening the Reporting of Observational Studies in Epidemiology (STROBE) guidance; the completed checklist is supplied separately (see Supporting Information).

## 3 Results

Participant characteristics are summarized in Table 1.

**TABLE 1:** Sociodemographic and descriptive characteristics.

	*n*	%
Gender		
Female	149	80.1
Male	37	19.9
Marital status		
Married	74	39.8
Single	112	60.2
Education status		
High school	36	19.3
Associate's degree	74	39.8
Bachelor's degree	58	31.2
Graduate degree	18	9.7
Income status		
Income lower than expenses	84	45.2
Income equals expenses	94	50.5
Income more than expenses	8	4.3
Liking the profession		
Yes	182	97.8
No	4	2.2
Work schedule		
Continuous daytime	83	44.6
On‐shift work	103	55.4
Clinical unit		
Surgical units	91	48.9
Internal units	37	19.9
Intensive care	24	12.9
Emergency service	34	18.3
Voluntarily chose current department		
Yes	167	89.8
No	19	10.2
Satisfaction with the clinical department		
Satisfied	169	90.9
Not satisfied	17	9.1
Recommending the profession to others		
Yes	156	83.9
No	30	16.1
Total	186	100

Other continuous variables				
	*M*	SD	Min	Max
Age (years)	29.69	7.77	19.00	55.00
Duration of employment in the institution	5.12	5.45	1 month	27 years
Duration of employment in the profession	8.42	7.34	1 month	28 years

Participants had a mean age of 29.69 years (SD 7.77); 80.1% were women, 60.2% were single, 55.4% worked shifts, and 48.9% worked in surgical units. Mean professional and institutional tenure were 8.42 years (SD 7.34) and 5.12 years (SD 5.45), respectively. Most participants had voluntarily chosen their current unit (89.8%), were satisfied with it (90.9%), liked the nursing profession (97.8%), and would recommend the profession to others (83.9%; Table 1). Scale descriptives and internal‐consistency estimates are presented in Table 2.

**TABLE 2:** Descriptive statistics on change fatigue and innovation climate scales and sub‐dimensions.

	Cronbach's *α*	*M*	SD	Median	Range
Change fatigue	0.836	2.85	0.77	3.00	1.00–5.00
Innovation climate	0.812	3.21	0.58	3.14	1.43–5.00
Support for innovation	0.886	3.47	0.82	3.60	1.00–5.00
Low barriers to innovation	0.846	3.09	0.86	3.00	1.00–5.00
Resource provision	0.756	2.99	0.88	3.00	1.00–5.00

Abbreviations: *M*, mean; range, observed minimum–maximum; SD, standard deviation.

The mean change fatigue score was 2.85 (SD 0.77), and the mean innovation climate score was 3.21 (SD 0.58). Among the innovation climate subscales, support for innovation had the highest mean (*M* 3.47, SD 0.82) and resource provision the lowest (*M* 2.99, SD 0.88). Cronbach's alpha was 0.836 for change fatigue and 0.812 for innovation climate; subscale values ranged from 0.756 to 0.886 (Table 2).

Group comparisons for change fatigue, innovation climate and its subscales are presented in Table 3.

**TABLE 3:** Scale scores according to selected participant and work characteristics.

Characteristic	Change fatigue	Innovation climate	Support for innovation	Low barriers to innovation	Resource provision
Category, *n*	*M* (SD)	*M* (SD)	*M* (SD)	*M* (SD)	*M* (SD)
Income < expenses, *n* = 84	2.91 (0.75)	3.02 (0.55)	3.19 (0.83)	3.03 (0.84)	2.70 (0.84)
Income = expenses, *n* = 94	2.86 (0.77)	3.34 (0.54)	3.71 (0.70)	3.08 (0.86)	3.24 (0.81)
Income > expenses, *n* = 8	2.13 (0.59)	3.61 (0.77)	3.58 (1.23)	3.88 (0.77)	3.13 (1.10)
Test; *p*	*F* = 3.936; 0.021	*F* = 9.716; < 0.001	*W* = 9.773; 0.001	*F* = 3.634; 0.028	*F* = 9.383; < 0.001
Day work, *n* = 83	2.66 (0.82)	3.31 (0.66)	3.47 (0.90)	3.35 (0.84)	2.97 (0.98)
Shift work, *n* = 103	3.00 (0.69)	3.12 (0.49)	3.47 (0.76)	2.88 (0.82)	3.00 (0.79)
Test; *p*	*t* = −3.033; 0.003	*t* = 2.249; 0.026	*t* = 0.039; 0.969	*t* = 3.842; < 0.001	*t* = −0.261; 0.795
Chose unit: yes, *n* = 167	2.82 (0.78)	3.23 (0.60)	3.50 (0.83)	3.09 (0.88)	3.04 (0.89)
Chose unit: no, *n* = 19	3.11 (0.57)	3.03 (0.30)	3.26 (0.75)	3.09 (0.73)	2.54 (0.59)
Test; *p*	*t* = −1.606; 0.110	*t* = 2.313; 0.026	*t* = 1.171; 0.243	*t* = 0.015; 0.988	*t* = 2.377; 0.019
Satisfied with unit: yes, *n* = 169	2.82 (0.76)	3.25 (0.58)	3.53 (0.81)	3.10 (0.86)	3.06 (0.86)
Satisfied with unit: no, *n* = 17	3.09 (0.78)	2.81 (0.47)	2.92 (0.79)	2.96 (0.90)	2.31 (0.78)
Test; *p*	*t* = −1.361; 0.175	*t* = 3.041; 0.003	*t* = 2.981; 0.003	*t* = 0.650; 0.516	*t* = 3.439; 0.001
Recommend profession: yes, *n* = 156	2.82 (0.78)	3.27 (0.58)	3.56 (0.81)	3.13 (0.88)	3.08 (0.87)
Recommend profession: no, *n* = 30	2.99 (0.65)	2.87 (0.45)	3.04 (0.73)	2.91 (0.76)	2.51 (0.76)
Test; *p*	*t* = −1.147; 0.253	*t* = 3.553; < 0.001	*t* = 3.224; 0.001	*t* = 1.247; 0.214	*t* = 3.365; 0.001

*Note:* Values are mean (SD). The six innovation‐barrier items were reverse scored; higher scores in the ‘low barriers’ column therefore indicate fewer perceived barriers to innovation. All *p* values are two‐sided.

Abbreviations: *F*, one‐way ANOVA; *t*, independent‐samples *t*‐test; *W*, Welch ANOVA.

Income groups differed in change fatigue and all innovation climate scores. Shift‐working nurses reported higher change fatigue and lower overall innovation climate and low‐barriers scores than day‐working nurses. Overall innovation climate and resource provision were higher among nurses who had voluntarily chosen their unit; overall innovation climate, support and resource provision were also higher among nurses satisfied with their unit and among those who would recommend the profession. No statistically significant differences in change fatigue were observed for voluntary unit choice, unit satisfaction, or recommendation of the profession (Table 3).

Pearson correlations among age, tenure, change fatigue and innovation climate are presented in Table 4.

**TABLE 4:** Pearson correlations among age, tenure, change fatigue and innovation climate.

Variables		1	2	3	4	5
1. Age	*r*	**1.000**	**0.897** **	**0.681** **	−0.023	0.139
	*p*		< 0.001	< 0.001	0.752	0.058
2. Duration of Employment in the Profession	*r*		**1.000**	**0.715** **	−0.039	**0.160** *
	*p*			< 0.001	0.602	**0.030**
3. Duration of Employment in the Institution	*r*			**1.000**	0.107	−0.015
	*p*				0.145	0.842
4. Change Fatigue Scale	*r*				**1.000**	**−0.438** **
	*p*					< 0.001
5. Innovation Climate Scale	*r*					**1.000**
	*p*					

*Note:* Two‐sided Pearson correlations.

* *p* < 0.05.

** *p* < 0.01.

Change fatigue and innovation climate were inversely correlated (*r* = −0.438, 95% CI [−0.547, −0.314], *p* < 0.001; *r* ^2^ = 0.192). Longer professional tenure was weakly correlated with higher innovation climate scores (*r* = 0.160, *p* = 0.030). Age and institutional tenure were not significantly correlated with either scale score (Table 4).

In the adjusted regression model, higher innovation climate remained associated with lower change fatigue (*B* = −0.528, 95% CI [−0.711, −0.345], standardized *β* = −0.400, *p* < 0.001). The full model explained 28.1% of the variance (adjusted *R* ^2^ = 0.253; *F*[7, 178] = 9.931, *p* < 0.001). Adding innovation climate increased *R* ^2^ by 0.131 (*F* change [1, 178] = 32.422, *p* < 0.001; incremental *f* ^2^ = 0.182). Diagnostics supported the model (maximum VIF = 2.09; Breusch–Pagan *p* = 0.544; maximum Cook's *D* = 0.080). The HC3 sensitivity estimate was materially unchanged (*B* = −0.528, 95% CI [−0.715, −0.341], *p* < 0.001; Table 5).

**TABLE 5:** Hierarchical multiple linear regression of change fatigue.

Predictor	*B*	SE	95% CI	Standardized *β*	*t*	*p*
Innovation climate	−0.528	0.093	−0.711, −0.345	−0.400	−5.694	< 0.001
Age (years)	−0.003	0.009	−0.021, 0.015	−0.029	−0.314	0.754
Male (ref: female)	−0.370	0.129	−0.625, −0.115		−2.862	0.005
Income = expenses	0.065	0.110	−0.153, 0.283		0.588	0.557
Income > expenses	−0.474	0.256	−0.979, 0.032		−1.850	0.066
Shift work (ref: day)	0.280	0.107	0.069, 0.491		2.619	0.010
Institutional tenure (years)	0.027	0.013	0.001, 0.052	0.191	2.081	0.039

*Note:* *N* = 186. Reference categories were female, income lower than expenses and day work. Model *R* ^2^ = 0.281; adjusted *R* ^2^ = 0.253.

Abbreviations: *B*, unstandardized coefficient; CI, confidence interval.

## 4 Discussion

In this two‐centre sample, nurses reported mean item scores near the midpoint for change fatigue and above the midpoint for perceived innovation climate. The principal finding was a consistent inverse association: nurses who perceived a more supportive innovation climate also tended to report less change fatigue. This association remained after adjustment for age, gender, income, work schedule and institutional tenure. Because exposure and outcome were measured at the same time, the result does not show whether climate perceptions preceded fatigue, whether fatigue influenced climate perceptions, or whether both reflected unmeasured organizational conditions.

The finding is consistent with the JD–R model, in which repeated change can be understood as a demand and innovation‐related support, autonomy, time, and resources as potential job resources (Demerouti et al. 2001). Adding innovation climate to the covariate model explained a further 13.1% of the variance in change fatigue (incremental *f* ^2^ = 0.182). This pattern supports the theoretical relevance of organizational resources, but a cross‐sectional regression cannot test a buffering mechanism or intervention effect.

The mean change fatigue score was lower than the high levels reported by Abdelwahid and Ata (2022). Differences in organizational change exposure, staffing, clinical workload, instruments and scoring may contribute to variation across studies. In the present sample, shift‐working nurses reported more change fatigue than day‐working nurses, consistent with evidence that shift patterns can be associated with fatigue and reduced recovery (Hong et al. 2021). Income groups also differed, as reported in another nursing study (Yu et al. 2025), although the group reporting income greater than expenses was small (*n* = 8); this estimate should therefore be interpreted cautiously.

No statistically significant differences in change fatigue were observed by marital status, education, clinical unit, voluntary unit choice, unit satisfaction, liking the profession or recommending nursing to others. Prior findings for demographic variables have also been mixed (Abdelwahid and Ata 2022; Elhay et al. 2022). These null group comparisons should not be interpreted as proof that such characteristics are irrelevant; several categories were small, and the study was not designed primarily for subgroup detection.

Innovation climate research in nursing has generally focused on innovative work behaviour, psychological empowerment, leadership, professional autonomy and factors associated with climate perceptions (Korku and Kaya 2023; Lv et al. 2021; Sun et al. 2024; Wang et al. 2024). The present analysis extends that literature by linking innovation climate to a change‐specific strain construct and by reporting both an unadjusted correlation and a confounder‐adjusted estimate. The association may reflect shared perceptions of communication, participation, managerial support and access to resources during change.

For nursing management, the results identify organizational conditions that merit structured assessment. During change initiatives, managers can monitor nurses' workload, communication quality, opportunity to participate and access to time and material resources. These actions are reasonable practice considerations, but their capacity to reduce change fatigue must be evaluated prospectively rather than assumed from the present association.

Theoretically, the findings support treating innovation climate as a multidimensional organizational resource rather than solely as a driver of innovation output. Future longitudinal studies can test temporal ordering, and cluster‐randomized or quasi‐experimental studies can evaluate whether strengthening specific resources changes fatigue while accounting for unit‐ and institution‐level context.

### 4.1 Strengths and Limitations

A strength of this study was the 95.9% response rate and the availability of complete item‐level data. Several limitations remain. First, the cross‐sectional design precludes temporal and causal inference. Second, self‐reported measures collected in one questionnaire are susceptible to common‐method and social‐desirability bias. Third, participants came from two healthcare centres in one region; organizational culture, staffing models, and management structures may differ across institutions, health systems, countries and nursing populations, which limits transferability. Site identifiers were not available in the analysis data, so institution‐level clustering could not be modelled. Fourth, the five‐point Change Fatigue Scale response format used in this study differs from the seven‐point format in the Turkish healthcare‐worker validation study, which may limit direct score comparability despite acceptable internal consistency in this sample. Finally, residual confounding is possible because change exposure, staffing ratios, workload, leadership and unit‐level resources were not measured directly.

## 5 Conclusion

Among nurses in two healthcare centres, higher perceived innovation climate was associated with lower change fatigue, and the association persisted after adjustment for selected demographic and work characteristics. The study does not establish that innovation climate reduces change fatigue.

The findings are relevant to nursing because repeated organizational change is implemented through nurses' daily work. Communication, meaningful participation, manageable workload and access to innovation resources are appropriate targets for organizational assessment and future prospective evaluation.

### 5.1 Implications for Nursing Management and Practice

Nursing leaders should communicate the rationale, timeline and expected workflow consequences of change; invite nurses' input early; and document whether staff have sufficient training, time and resources. These steps may improve implementation conditions, but the present study cannot determine their effect on fatigue.

Organizations can also track change fatigue and innovation‐climate indicators alongside staffing, workload, retention and patient‐safety measures. Repeated measurement would help identify units requiring support and would generate stronger temporal evidence than a single cross‐sectional assessment.

Future multicentre studies should include varied health systems and nursing groups, measure organizational change exposure directly, and distinguish nurse‐, unit‐ and institution‐level effects. Intervention studies are needed before causal practice recommendations are made.

## Author Contributions

**Yeter Uslu:** conceptualization, supervision, writing – original draft, writing – review and editing. **Yasemin Hancioğlu Başköy:** conceptualization, supervision, writing – original draft, writing – review and editing. **Gözde Yeşilaydin:** conceptualization, formal analysis, supervision, writing – original draft. **Erman Gedikli:** investigation, formal analysis, supervision, writing – original draft. **Emre Yilmaz:** investigation, formal analysis, supervision, writing – original draft.

## Funding

The Article Processing Charge (APC) for the publication of this article was funded by Istanbul Medipol University.

## Ethics Statement

Ethical approval was obtained from the Istanbul Medipol University Non‐Interventional Clinical Research Ethics Committee (approval no. E‐10840098‐772.02‐1544; March 4, 2022).

## Consent

All participants received information about the study and provided written informed consent before participation.

## Conflicts of Interest

The authors declare no conflicts of interest.

## Supporting information


**Data S1:** Completed STROBE checklist for this cross‐sectional study.

## Data Availability

Data available on request from the authors.
